# Case Report: Artifactual Hypoglycemia: A Condition That Should Not Be Forgotten

**DOI:** 10.3389/fendo.2022.951377

**Published:** 2022-07-28

**Authors:** Sara Amaral, Ana Palha, Vera Bernardino, José Silva-Nunes

**Affiliations:** ^1^Department of Endocrinology, Diabetes and Metabolism; Centro Hospitalar Universitário Lisboa Central, Lisbon, Portugal; ^2^Nova Medical School/Faculdade de Ciencias Medicas, Universidade Nova de Lisboa, Lisbon, Portugal; ^3^Functional Unit of Internal Medicine 7.2, Centro Hospitalar Universitário Lisboa Central, , Lisbon, Portugal; ^4^Health and Technology Research Center (H&TRC), Escola Superior de Tecnologia da Saude de Lisboa, Lisbon, Portugal

**Keywords:** hypoglycemia, raynaud’s phenomenon, capillary blood glucose, artifactual hypoglycemia, autoimmune diseases

## Abstract

**Background:**

Hypoglycemia is uncommon in people who are not being treated for diabetes mellitus and, when present, the differential diagnosis is broad. Artifactual hypoglycemia describes discrepancy between low capillary and normal plasma glucose levels regardless of symptoms and should be considered in patients with Raynaud’s phenomenon.

**Case Presentation:**

A 46-year-old female patient with a history of a sleeve gastrectomy started complaining about episodes of lipothymias preceded by sweating, nausea, and dizziness. During one of these episodes, a capillary blood glucose was obtained with a value of 24 mg/dl. She had multiple emergency admissions with low-capillary glycemia. An exhaustive investigation for possible causes of hypoglycemia was made for 18 months. The 72h fasting test was negative for hypoglycemia. A Raynaud’s phenomenon was identified during one appointment.

**Conclusion:**

Artifactual hypoglycemia has been described in various conditions including Raynaud’s phenomenon, peripheral arterial disease, Eisenmenger syndrome, acrocyanosis, or hypothermia. With this case report, we want to reinforce the importance of being aware of this diagnosis to prevent anxiety, unnecessary treatment, and diagnostic tests.

## Introduction

Hypoglycemia usually occurs in the setting of the treatment of glucose-lowering agents such as sulfonylureas or insulin (1). The diagnosis of hypoglycemia requires fulfillment of Whipple’s triad: symptoms and/or signs consistent with hypoglycemia, presence of a low plasma glucose concentration at the time of the suspected hypoglycemia, and resolution of symptoms or signs when hypoglycemia is corrected (2). The investigation of the hypoglycemia cause should only be initiated after fulfillment of Whipples’s triad. Hypoglycemia is uncommon in people who are not being treated for diabetes mellitus and, when present, the differential diagnosis is broad. Drugs, critical illnesses, hormone deficiencies, and non-islet cell tumors should be considered in those who are ill or taking medications. Other causes include endogenous hyperinsulinism due to insulinoma, functional β cell disorders, or insulin autoimmune conditions. Hypoglycemia can also occur after bariatric surgery (3). However, there are some reports of artifactual hypoglycemia. This term has been proposed to describe discrepancy between low capillary and normal plasma glucose levels regardless of symptoms (4). We present a clinical report of artifactual hypoglycemia in a patient with Raynaud’s phenomenon and a previous history of bariatric surgery.

## Case Presentation

A 46-year-old female patient was referred to the Department of Endocrinology, Diabetes and Metabolism due to suspected hypoglycemia. She had history of dyslipidemia and had lost 50 kg after being submitted to sleeve gastrectomy in 2016. She was under therapy with omeprazole 20 mg/day and simvastatin 20 mg/day. She had no family history of diabetes. About 18 months after sleeve gastrectomy, she started complaining about episodes of lipothymias preceded by sweating, nausea, and dizziness. During one of these episodes, a capillary blood glucose was obtained with a value of 24 mg/dl. After this episode, she had several admissions to the emergency department with the diagnosis of “hypoglycemia” after capillary measuring blood glucose at home. In this setting, she was admitted several times to another hospital to clarify her clinical picture. The performed blood tests relative to glucose metabolism are reported in **Table 1**. The remaining blood tests did not show any changes including normal blood count, kidney function, liver enzymes, thyroid function, and calcium. Different imaging exams were performed including abdominal computed tomography (CT), abdominal magnetic resonance (MRI), upper endoscopy, endoscopic ultrasound, fluorodeoxyglucose (FDG)–positron emission tomography (PET), and ^68^Ga-DOTANOC PET/CT. No changes were found.

**Table 1:** Blood test relative to glucose metabolism.

Blood test	14/08/2019	14/10/2019	29/11/2020
Glucose (mg/dl)	75		
Insulin (RV 3-25 mcUI/ml)	6.2		
Pro-insulin (RV <9.4 mcUI/ml)	<0.6		
C-peptide (ng/ml)	1.98		
72-h fasting test		No evidence of hypoglcyemia	
Oral glucose tolerance test			0′ – 74 mg/dl 60′ – 109 mg/dl 120′ – 61 mg/dl
Anti-GAD			Negative
Anti-insulin			Negative
Anti-ICA			Negative

Due to persistent complaints even after all the investigation, she was referred to the Department of Endocrinology, Diabetes and Metabolism of Centro Hospitalar Universitário Lisboa Central.

She had the first appointment in our department in December 2020. According to the patient, she needed to eat sugary foods or beverages every 2h during the daytime and twice during nighttime to solve symptoms. She was previously treated with acarbose due to suspected Dumping syndrome with no improvement. Her weight increased 6 kg in 1 year. She reported capillary glycemia between 23mg/dl and 45 mg/dl during those episodes.

**Figure 1:**
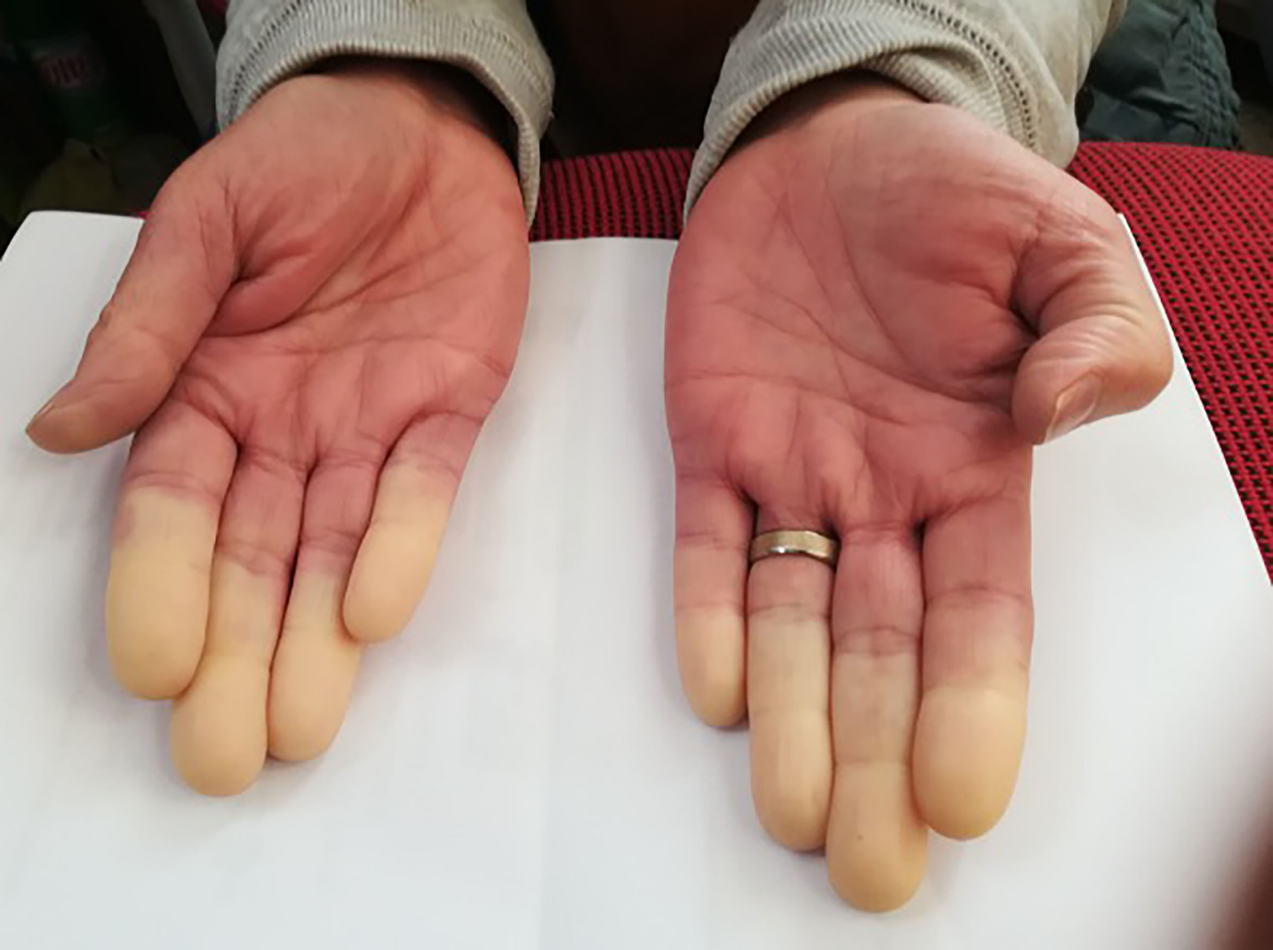
Raynaud’s phenomenon during endocrinology appointment.

On examination, the patient was alert and cooperative. Her weight was 82 kg and height was 1.65 m (body mass index of 30.1 kg/m^2^). Blood pressure was 147/81 mmHg and heart rate 78 bpm. She reported having had a coffee with sugar 1h before the appointment. A capillary blood glucose was obtained with a value of 54 mg/dl. At the same time, a first venous blood sample was obtained. After 15 min, she started complaining about palpitations, headache, and tremors. Vital signs were measured again with blood pressure 194/130 mmHg, heart rate 84 bpm, and capillary blood glucose 24 mg/dl. A Raynaud’s phenomenon (**Figure 1**) was observed. The remaining examination was unremarkable. The patient did not have any changes from her baseline neurological status during these episodes. A second venous blood sample was obtained; the results are displayed in **Table 2**. Hypoglycemia was not confirmed and a diagnosis of artifactual hypoglycemia was considered. Plasma-free metanephrines were in the normal range.

**Table 2:** Discrepancy between capillary and venous blood glucose.

	Capillary blood glucose	Plasma blood glucose
First venous blood sample	54 mg/dl	78 mg/dl
Second venous blood sample	24 mg/dl	76 mg/dl

The patient reported that Raynaud’s phenomenon was concomitant to the several episodes suggestive of hypoglycemia. She was advised to fractionate meals and to avoid fast-absorbing carbohydrates. An Autoimmune Disease appointment was requested.

The complementary study on the Autoimmune Disease appointment revealed HLA-B27 positivity and increased Erythrocyte Sedimentation Rate (ESR) 66 mm/h (<16). The remaining investigation was negative for rheumatoid factor, antinuclear (ANA), anti-dsDNA, anti-nucleosome, extractable nuclear antigen (ENA), including SSA, SSB, RNP/Sm, Sm, Jo-1, Scl-70, histones, and ribosomal-P. Anti-centromere B, anti-fibrillarin, anti-NOR 90, anti-TH/To, and anti-citrulline antibodies were also negative. The remaining laboratory results were unremarkable (haemoglobin 12.6 × 10 g/L (12.0–15.0), normal leukocyte count, blood urea nitrogen test 31 mg/dl (15.0–40.0), creatinine 0.74 mg/dl (0.57–1.11), estimated glomerular filtration rate 97 ml/min/1.73 and normal liver enzymes). She also presented lower limb dactylitis. Capillaroscopy revealed a secondary Raynaud's phenomenon with early scleroderma pattern. A presumptive diagnosis of ankylosing spondylitis was made while radiological studies are ongoing. At the last appointment (February 2022), she was medicated with amlodipine 5 mg/day, salazopyrine 3 g/day, and etoricoxib 90 mg as needed with symptoms improvement. No hypoglycemic episodes were observed after nutritional intervention.

## Ethics Consideration and Patient Details

Written informed consent was obtained from the patient for the publication of this case report and accompanying images.

## Discussion

Artifactual hypoglycemia has been described in various conditions including Raynaud’s phenomenon, peripheral arterial disease, Eisenmenger syndrome, acrocyanosis, or hypothermia (4). The Raynaud’s phenomenon was first described in the 19^th^ century as episodic, symmetrical, and vasospastic disorder, resulting in classic triphasic color change, trophic changes limited to the skin and uncomfortable sensory symptoms of the extremities in the absence of arterial occlusion (5). In this situation, there is a reduced perfusion of the peripheral microcirculation with decelerated glucose transit and increased glucose uptake into the surrounding tissues (6). Other conditions such as leukemia could also lead to artifactual hypoglycemia due to increased glycolysis by leukocytes (7).

During more than 1 year, this patient had several hospitalizations to investigate possible causes of hypoglycemia, which illustrates the importance of awareness for artifactual hypoglycemia. The patient’s belief of having a medical condition that was responsible for the hypoglycemia was difficult to deconstruct. Even after the confirmation of the diagnosis and after all the explanations, she kept measuring capillary blood glucose at home.

The patient’s symptoms initially attributed to hypoglycemia were probably justified by a dumping syndrome and improved with dietary education.

With this case report, we want to reinforce the importance of being aware of this diagnosis to prevent anxiety, unnecessary treatment, and diagnostic tests. In patients with suspected artifactual hypoglycemia, using ear lobe pricks for the assessment of blood glucose can improve the accuracy of the results in this setting (8).

## Data Availability Statement

The raw data supporting the conclusions of this article will be made available by the authors, without undue reservation.

## Ethics Statement

Written informed consent was obtained from the individual(s) for the publication of any potentially identifiable images or data included in this article.

## Author Contributions

All the authors have contributed significantly. SA designed the study. SA and VB wrote the manuscript. AP and JS-N supervised the study and corrected the manuscript. All authors contributed to the article and approved the submitted version.

## Funding

This research did not receive any specific grant from any funding agency in the public, commercial, or not-for-profit sector.

## Conflict of Interest

The authors declare that the research was conducted in the absence of any commercial or financial relationships that could be construed as a potential conflict of interest.

## Publisher’s Note

All claims expressed in this article are solely those of the authors and do not necessarily represent those of their affiliated organizations, or those of the publisher, the editors and the reviewers. Any product that may be evaluated in this article, or claim that may be made by its manufacturer, is not guaranteed or endorsed by the publisher.

## References

[1] J. &. G. P. Guettier. Hypoglycemia. Endocrinol Metab Clinics North America (2006) 35:753–66. doi: 10.1016/j.ecl.2006.09.005 17127144

[2] Kittah NE Vella A. Pathogenesis and Management of Hypoglycemia. Eur Journ Endoc (2017) 177:R37–47. doi: 10.1530/EJE-16-1062 28381450

[3] Cryer P Axelrod L Grossman A Heller S Montori V Seaquist E. Evaluation and Management of Adult Hypoglycemic Disorders: An Endocrine Society Clinical Practice Guideline. J Clin Endocrinol Metab (2009) 94:709–28. doi: 10.1210/jc.2008-1410 19088155

[4] Tarasova V Zena M Rendell M. Artifactual Hypoglycemia: An Old Term for a New Classification. Diabetes Care (2014) 37:e85–6. doi: 10.2337/dc13-2891 24757247

[5] Bowling J Dowd P. Raynaud’s Disease. Lancet (2003) 361:2078–80. doi: 10.1016/S0140-6736(03)13646-X 12814733

[6] Drenthen L Verheggen R Galan B. Clinical Impact of Artifactual Hypoglycaemia and its Diagnosis at the Bedside. Rheumatology (2019) 58(9):1691–2. doi: 10.1093/rheumatology/kez118 PMC6736385 31211383

[7] Bishay R Suryawanshi A. Artifactual Hypoglycaemia in Systemic Sclerosis and Raynaud’s Phenomenon: A Clinical Case Report and Short Review. Case Rep Endocrinol (2016) 2016:7390927. doi: 10.1155/2016/7390927 28116181 PMC5225329

[8] Garner R Kumari R Lanyon P Doherty M Zhang W. Prevalence, Risk Factors and Associations of Primary Raynaud’s Phenomenon: Systematic Review and Meta-Analysis of Observational Studies. BMJ Open (2015) 5(3):e006389. doi: 10.1136/bmjopen-2014-006389 PMC4368987 25776043

